# Validation of Left Atrial Volume Correction for Single Plane Method on Four-Chamber Cine Cardiac MRI

**DOI:** 10.3390/tomography10040035

**Published:** 2024-03-25

**Authors:** Hosamadin Assadi, Nicholas Sawh, Ciara Bailey, Gareth Matthews, Rui Li, Ciaran Grafton-Clarke, Zia Mehmood, Bahman Kasmai, Peter P. Swoboda, Andrew J. Swift, Rob J. van der Geest, Pankaj Garg

**Affiliations:** 1 Department of Cardiovascular and Metabolic Health, Norwich Medical School, University of East Anglia, Norwich NR4 7TJ, UK; 2 Department of Cardiology, Norfolk and Norwich University Hospitals NHS Foundation Trust, Norwich NR4 7UY, UK; 3 Faculty of Medicine, Medical University of Sofia, Blvd Akademik Ivan Evstratiev Geshov 15, 1431 Sofia, Bulgaria; 4 Division of Biomedical Imaging, Leeds Institute of Cardiovascular and Metabolic Medicine, University of Leeds, Leeds LS2 9JT, UK; 5 Department of Infection, Immunity & Cardiovascular Disease, University of Sheffield, Sheffield S10 2RX, UK; 6 Department of Radiology, Division of Image Processing, Leiden University Medical Center, 2333 ZA Leiden, The Netherlands

**Keywords:** heart atria, magnetic resonance imaging, CMR, image processing, atrial function

## Abstract

Background: Left atrial (LA) assessment is an important marker of adverse cardiovascular outcomes. Cardiovascular magnetic resonance (CMR) accurately quantifies LA volume and function based on biplane long-axis imaging. We aimed to validate single-plane-derived LA indices against the biplane method to simplify the post-processing of cine CMR. Methods: In this study, 100 patients from Leeds Teaching Hospitals were used as the derivation cohort. Bias correction for the single plane method was applied and subsequently validated in 79 subjects. Results: There were significant differences between the biplane and single plane mean LA maximum and minimum volumes and LA ejection fraction (EF) (all *p* < 0.01). After correcting for biases in the validation cohort, significant correlations in all LA indices were observed (0.89 to 0.98). The area under the curve (AUC) for the single plane to predict biplane cutoffs of LA maximum volume ≥ 112 mL was 0.97, LA minimum volume ≥ 44 mL was 0.99, LA stroke volume (SV) ≤ 21 mL was 1, and LA EF ≤ 46% was 1, (all *p* < 0.001). Conclusions: LA volumetric and functional assessment by the single plane method has a systematic bias compared to the biplane method. After bias correction, single plane LA volume and function are comparable to the biplane method.

## 1. Introduction

The importance of the left atrium (LA) structure and function in predicting cardiovascular health outcomes is well established. For instance, an enlarged LA size has been linked to a higher risk of cardiovascular events in the general population, as well as a poorer prognosis for patients with heart failure and myocardial infarction [1,2,3,4]. Furthermore, an enlarged LA size has been associated with the onset of atrial fibrillation (AF) and stroke [5,6,7]. Similarly, diminished LA function correlates with cardiovascular events in the general population and in patients with preserved left ventricular (LV) systolic function [8]. In clinical practice, cardiovascular magnetic resonance (CMR) offers precise and reproducible LA structure and function measurements with high spatial and temporal resolution without the risk of radiation exposure [9,10]. The American College of Cardiology Foundation and the American Heart Association recommend using noninvasive imaging modalities, including CMR, when transthoracic echocardiography is unable to evaluate cardiac structure and function accurately [11,12]. LA structure and functional assessment are mainly done using biplane long-axis views, including four-chamber and two-chamber views [13,14]. In addition, more recently, the CMR assessment of LA has been shown to be important in assessing left ventricular filling pressure (LVFP) and hemodynamics as it can measure acute and dynamic changes in preloading conditions on the left ventricle [15]. CMR can estimate LVFP in patients with suspected heart failure, and CMR-modelled LVFP has been shown to have prognostic power [15,16].

To streamline the workflow in daily clinical practice, quantifying LA function and structure from a single plane may be sufficient [17]. However, the accuracy of these measurements from a single plane method is yet to be determined. In addition, sometimes, due to temporal differences in four-chamber and two-chamber cines, errors can be introduced in quantifying LA volume and function [18]. Finally, occasionally, some patients do not complete their CMR examination due to claustrophobia, which can further limit the application of the biplane method. Hence, an alternative single plane four-chamber method would be desirable for LA quantification.

We hypothesize that four-chamber imaging alone can provide accurate and reliable measurements of LA volumes and function.

## 2. Materials and Methods

### 2.1. Study Cohort

This study consisted of a cohort of 179 patients with several cardiovascular diseases who have been previously prospectively recruited to other studies between 2014 to 2018 [19,20]. The group was divided into derivation (*n* = 100) and validation (*n* = 79) cohorts. The inclusion criteria specific to this study were individuals over the age of 18 who provided written informed consent. In addition, a four-chamber, well-planned cine and two-chamber cine were needed for the patients to be enrolled to this work. The exclusion criteria were estimated glomerular filtration rate (eGFR) < 30 mL/min/1.73 m^2^, claustrophobia and any CMR contraindications.

### 2.2. Ethics Approval and Consent to Participate

The research adhered to the guidelines outlined in the 2013 version of the Declaration of Helsinki. Data acquisition and handling were authorised by the National Research Ethics Service in the UK, with approval number 21/NE/0149.

### 2.3. Cardiac Magnetic Resonance Protocol

The CMR images for this study were performed on a 1.5 T Ingenia system by Philips Healthcare (Best, The Netherlands) equipped with a Biometric body with 28-channel coils and digitisation of the CMR signal in the receiver coil. The CMR protocol included baseline survey images, cines (vertical long-axis, horizontal long-axis, 3-chamber and the LV volume contiguous short axis stack), early and late gadolinium enhancement imaging, two-dimensional phase contrast and four-dimensional flow acquisition methods previously described by our group [19,20]. Following planning sequences, four-chamber cine images were acquired, followed by a stack of short-axis cine images covering apex to base. For standard cines, we acquired 30 phases throughout the cardiac cycle. Other cine acquisition parameters include an 8-mm slice thickness, repetition time of 2.72 ms and echo time of 1.36 ms using a standard cardiac-gated balanced steady-state free precession (bSSFP) sequence.

### 2.4. CMR Analysis

All analyses used MASS research software (version 2023—EXP, Leiden University Medical Center, Leiden, The Netherlands). Endocardial contours for the left and right ventricle and atria structures were drawn in all cardiac phases using artificial intelligence models previously described [21] in the four-chamber cine. Epicardial contours were also drawn for the left ventricle. Endocardial contours for the left ventricle and left atria structures were drawn for the two-chamber cine. For quality control, the AI-generated segmentations and time-resolved volume curves throughout all cardiac phases for all four chambers were evaluated by one of the authors (HA) and double-checked by an experienced clinician (SCMR Level 3).

### 2.5. Statistical Analysis

Data were analysed using MedCalc^®^ Statistical Software, version 22.014 (MedCalc Software Ltd., Ostend, Belgium). Continuous variables were expressed as the mean ± standard deviation (SD). Discrete data were presented as numbers (n) and percentages (%). All data were treated as parametric. Variables were compared between groups using an independent sample t-test. Correlations between the two methods were evaluated using Pearson’s coefficient of rank correlation (r). We used Bland Altman plot analysis to check for agreement and bias between the methods. The discriminative capability was evaluated by calculating the area under the curve (AUC) using receiver operating characteristic (ROC) curves. The statistical significance was set at *p* < 0.05.

## 3. Results

### 3.1. Left Atrial Biplanar Volume Assessment in the Whole Cohort

**Figure 1:**
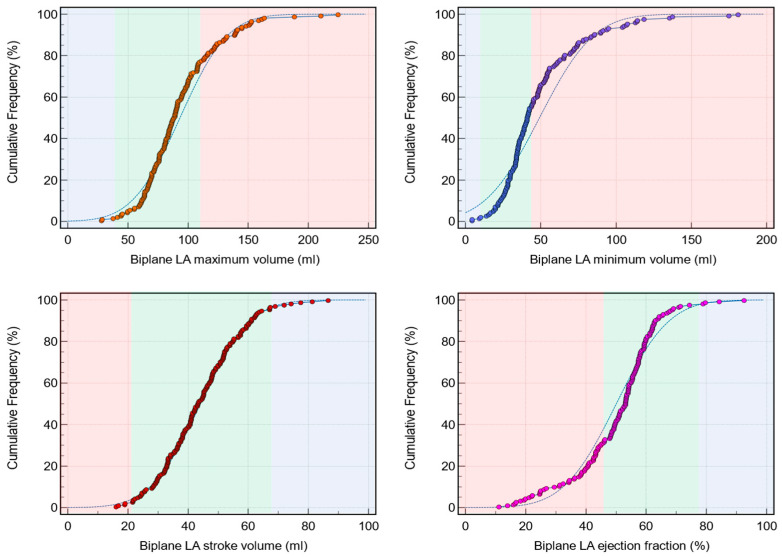
Cumulative frequency distribution of biplane left atrial (LA) parameters in the whole study cohort (*n* = 179). Each graph’s two distinct background colors divide them at the halfway point of the *y*-axis, indicating different zones or reference ranges for easier visual interpretation. The figure effectively visualizes the distributions of the biplane LA parameters measured in the study.

In total, we enrolled 179 patients. Figure 1 demonstrates the broad cumulative distribution of LA volume and functional metrics across all normal and abnormal values ranges. In the total cohort, 40 patients (22%) had elevated LA maximum volume (≥112 mL). For LA minimum volume, 79 patients (44%) had high LA minimum volume (≥44 mL). Only four patients (2%) had reduced LA SV (≤21 mL). However, 56 patients (31%) had reduced LA EF (≤46%).

For the LA maximum volume, the biplane method showed a range of 28–225 mL, with a mean of 93 mL (95% CI: 89–98 mL), median of 88 mL (95% CI: 84–91 mL), and standard deviation of 31 mL. The relative standard deviation was 34%, and the standard error was 2.3 mL. For the LA minimum volume, the range was 4.5–181 mL, with a mean of 49 mL (95% CI: 45–53 mL), median of 41 mL (95% CI: 38–45 mL), and standard deviation of 29 mL. The relative standard deviation was 58%, and the standard error was 2 mL. For the LA SV, the biplane method showed a range of 16–87 mL, with a mean of 44 mL (95% CI: 42–46 mL), median of 43 mL (95% CI: 41–46 mL), and standard deviation of 13 mL. The relative standard deviation was 30%, and the standard error was 1 mL. For the LA EF, the range was 11–93%, with a mean of 50% (95% CI: 48–52%), median of 53% (95% CI: 51–54%), and standard deviation of 14%. The relative standard deviation was 27%, and the standard error was 1%.

### 3.2. Derivation Cohort (n = 100)

#### 3.2.1. Correlations between Biplanar and Single Planar Methods

**Figure 2:**
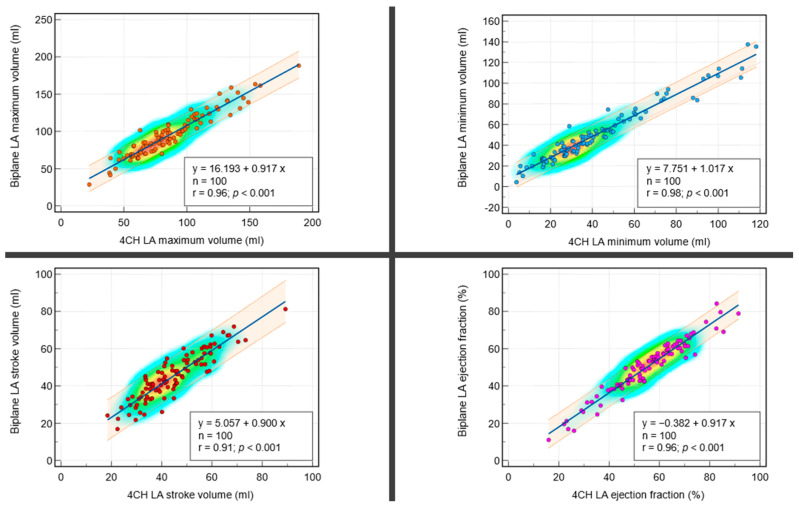
Scatter plots with heat maps demonstrating correlations between single plane four-chamber cine (4CH) left atrial (LA) parameters and biplane LA parameters. The color-coding of data points represents data density, with red indicating higher density and blue signifying lower density. Each plot includes a linear regression line with confidence intervals, demonstrating the strength and direction of the correlations. The figure effectively visualizes the strong positive correlations between the single plane and biplane LA parameters measured in the study.

The regression equations and scatter plots are presented in Figure 2. The regression analysis of the dependent variable biplane maximum LA volume (in mL) on the independent variable single plane maximum LA volume (in mL) yielded a highly significant model (*p* < 0.001) with a strong coefficient of determination (r = 0.96), indicating that approximately 96% of the variation in biplane maximum LA volume can be explained by single plane maximum LA volume. The regression equation is y = 16.193 + 0.917x. The intercept is 16.2 (95% CI: 11.0 to 21.3, t = 6.2, *p* < 0.001), and the slope is 0.9 (95% CI: 0.9 to 1.0, t = 32.4, *p* < 0.001), both of which are statistically significant.

The regression analysis of the dependent variable biplane minimum LA volume (in mL) on the independent variable single plane minimum LA volume (in mL) yielded a highly significant model (*p* < 0.001) with a strong coefficient of determination (r = 0.98), indicating that approximately 98% of the variation in biplane minimum LA volume can be explained by single plane minimum LA volume. The regression equation is y = 7.751 + 1.017x. The intercept is 7.8 (95% CI: 5.5 to 10.0, t = 6.9, *p* < 0.001), and the slope is 1.0 (95% CI: 0.97 to 1.06, t = 44.2, *p* < 0.001), both of which are statistically significant.

The regression analysis of the dependent variable biplane LA SV (in mL) on the independent variable single plane LA SV (in mL) yielded a significant model (*p* < 0.001) with a strong coefficient of determination (r = 0.91), indicating that approximately 91% of the variation in biplane LA SV can be explained by single plane LA SV. The regression equation is y = 5.057 + 0.900x. The intercept is 5.1 (95% CI: 1.2 to 8.9, t = 2.6, *p* = 0.01), and the slope is 0.9 (95% CI: 0.82 to 0.98, t = 21.4, *p* < 0.001), both of which are statistically significant.

The regression analysis of the dependent variable biplane LA EF (in %) on the independent variable single plane LA EF (in %) yielded a significant model (*p* < 0.001) with a strong coefficient of determination (r = 0.96), indicating that approximately 96% of the variation in biplane LA EF can be explained by single plane LA EF. The regression equation is y = −0.382 + 0.917x. The intercept is −0.4 (95% CI: −3.2 to 2.5, t = −0.3, *p* = 0.79), and the slope is 0.9 (95% CI: 0.87 to 0.97, t = 36.3, *p* < 0.001), both of which are statistically significant.

#### 3.2.2. Bland–Altman Plots and Bias Investigation

**Figure 3:**
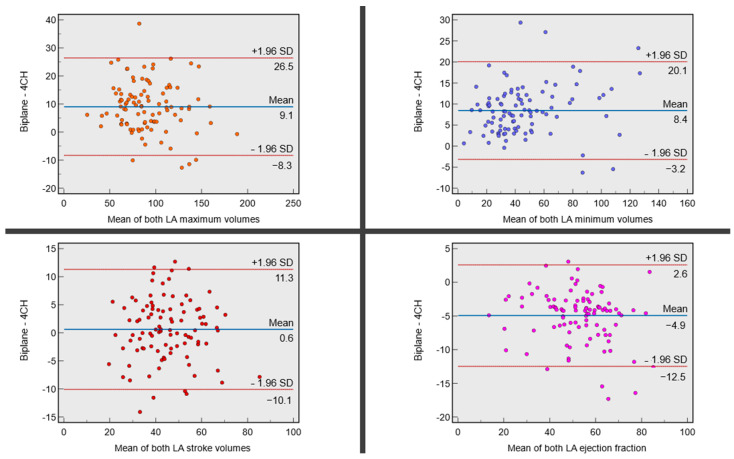
Bland–Altman plots for all left atrial (LA) variables demonstrate significant bias between biplane and single plane methods for LA maximum and minimum volumes. Both are systematically underestimated by the single plane method. Stroke volume (SV) is similar between both groups. Due to the relative difference in maximum and minimum volumes, the single plane method systematically overestimates the LA ejection fraction (EF). Other than for stroke volume, all *p*-values are significant for paired comparison.

In a derivation cohort of 100 subjects, the comparison between the biplane and single plane methods for measuring LA volume and function revealed significant differences (Table 1). The Bland–Altman plots are presented in Figure 3. The mean ± SD of maximum LA volume was found to be lower in the single plane method (86 ± 30 mL) compared to the biplane method (96 ± 29 mL), with a bias of −9 mL and a standard deviation of bias of 9 mL (*p* < 0.01). Similarly, the mean minimum LA volume was also lower in the single plane method (42 ± 26 mL) compared to the biplane method (50 ± 27 mL), with a bias of −8 mL and a standard deviation of bias of 6 mL (*p* < 0.01). However, there was no significant difference in the LA SV between the two methods (45 ± 13 mL for both), with a negligible bias of −1 mL and an SD of bias of 6 mL (*p* = 0.27). Interestingly, the mean LA EF was found to be higher in the single plane method (55 ± 15%) compared to the biplane method (50 ± 14%), with a bias of 5% and an SD of bias of 4% (*p* < 0.01). The SD of the bias was used to correct the systematic bias in these measurements.

### 3.3. Validation Cohort (n = 79)

#### 3.3.1. Left Atrial Volumetric Assessment

The arithmetic mean ± SD of the LA maximum volume derived using the biplane and single plane methods were (90 ± 34 mL, 95% CI: 82.8 to 97.9 mL) and (83 ± 34 mL, 95% CI: 75.8 to 90.8 mL), respectively, indicating that the true population mean is likely within this range. The median values for the biplane and single plane methods were (86 mL, 95% CI: 78.9 to 90.7 mL) and (76 mL, 95% CI: 71.4 to 86.3 mL), respectively. The variance, which is a measure of dispersion for biplane and single plane methods, was 1132 mL and 1127 mL, respectively. The relative standard deviation for the biplane method was 37%, and the single plane method was 34%, providing a measure of dispersion relative to the mean. The standard error of the mean, which estimates the precision of the sample mean as an estimate of the population mean, was 3.8 mL for both methods.

For LA minimum volume, the arithmetic mean ± SD using the biplane and single plane methods were (48 ± 30 mL, 95% CI: 40.9 to 54.6 mL) and (41 ± 30 mL, 95% CI: 34.6 to 47.9 mL), respectively, indicating that the true population mean is likely within this range. The median values for the biplane and single plane methods were (39 mL, 95% CI: 34.9 to 44.8 mL) and (32 mL, 95% CI: 28.5 to 38.5 mL), respectively. The variance, which is a measure of dispersion for the biplane and single plane methods, was 923 mL and 883 mL, respectively. The relative standard deviation for the biplane method was 64%, and the single plane method was 72%, providing a measure of dispersion relative to the mean. The standard error of the mean, which estimates the precision of the sample mean as an estimate of the population mean, was 3.4 mL and 3.3 mL for biplane and single plane methods, respectively.

The biplane and single plane LA SV’s arithmetic mean ± SD were (43 ± 14 mL, 95% CI: 39.6 to 45.7 mL) and (42 ± 13 mL, 95% CI: 39.3 to 44.9 mL), respectively, indicating that the true population mean is likely within this range. The median values for the biplane and single plane methods were (41 mL, 95% CI: 37.6 to 45.9 mL) and (41 mL, 95% CI: 38.9 to 45.2 mL), respectively. The variance, which is a measure of dispersion for the biplane and single plane methods, was 184 mL and 158 mL, respectively. The relative standard deviation for the biplane method was 32%, and the single plane method was 30%, providing a measure of dispersion relative to the mean. The standard error of the mean, which estimates the precision of the sample mean as an estimate of the population mean, was 1.5 mL and 1.4 mL for biplane and single plane methods, respectively.

The biplane and single plane method derived LA EF’s arithmetic mean ± SD were (50 ± 14%, 95% CI: 47 to 53.1%) and (54 ± 15%, 95% CI: 50.9 to 57.5%), respectively, indicating that the true population mean is likely within this range. The median values for the biplane and single plane methods were (54%, 95% CI: 50.5 to 55.8%) and (59%, 95% CI: 54.1 to 61%), respectively. The variance, which is a measure of dispersion for the biplane and single plane methods, was 187% and 213%, respectively. The relative standard deviation for both was 27%, providing a measure of dispersion relative to the mean. The standard error of the mean, which estimates the precision of the sample mean as an estimate of the population mean, was 1.5% for the biplane method and 1.6% for the single plane method.

#### 3.3.2. Correlations between Biplanar and Single-Planar after Bias Correction

**Figure 4:**
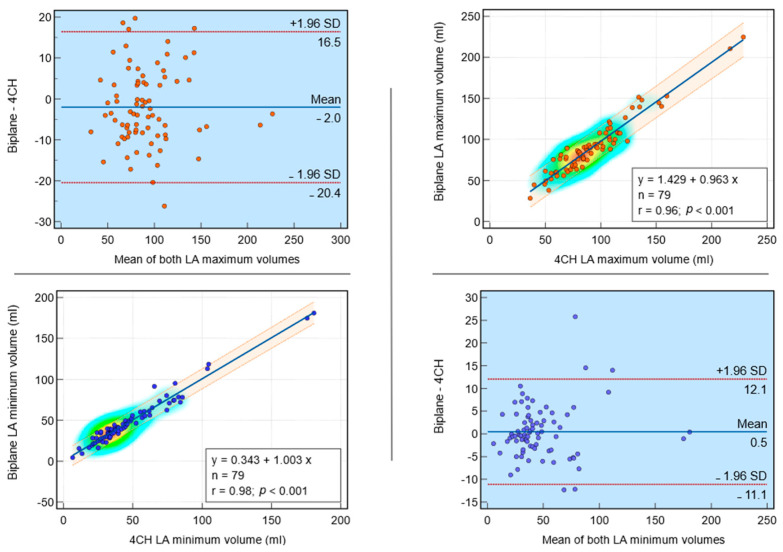
Post-bias correction agreement investigation in the validation cohort (*n* = 79): Bland–Altman plot agreement and correlation between biplane and single plane methods of left atrial (LA) maximum and minimum volumes assessment methods. Both Bland–Altman and regression analyses demonstrate the agreement of biplane versus single plane measurements for these two variables.

In a validation cohort of 79 subjects, the regression analysis of the dependent variable biplane LA maximum volume (in mL) on the independent variable single plane LA maximum volume (in mL) yielded a significant model (*p* < 0.001) with a strong coefficient of determination (r = 0.96). The regression equation was y = 1.429 + 0.963x. The intercept was 1.4 (95% CI: −4.8 to 7.6, t = 0.5, *p* = 0.65), and the slope was 1.0 (95% CI: 0.9 to 1.0, t = 30.4, *p* < 0.0001) (Figure 4). For biplane LA minimum volume (in mL) and single plane LA minimum volume (in mL), the regression equation was y = 0.343 + 1.003x with a strong coefficient of determination (r = 0.98, *p* < 0.001). For biplane LA EF (in %) and single plane LA EF (in %), the regression equation was y = 4.617 + 0.905x with a strong coefficient of determination (r = 0.97, *p* < 0.001). For biplane LA SV (in mL) and single plane LA SV (in mL), the regression equation was y = 2.397 + 0.955x with a strong coefficient of determination (r = 0.89, *p* < 0.001). These findings suggest a strong positive linear relationship between each pair’s dependent and independent variables.

#### 3.3.3. Bland–Altman Plots and Bias Investigation after Bias Correction

**Figure 5:**
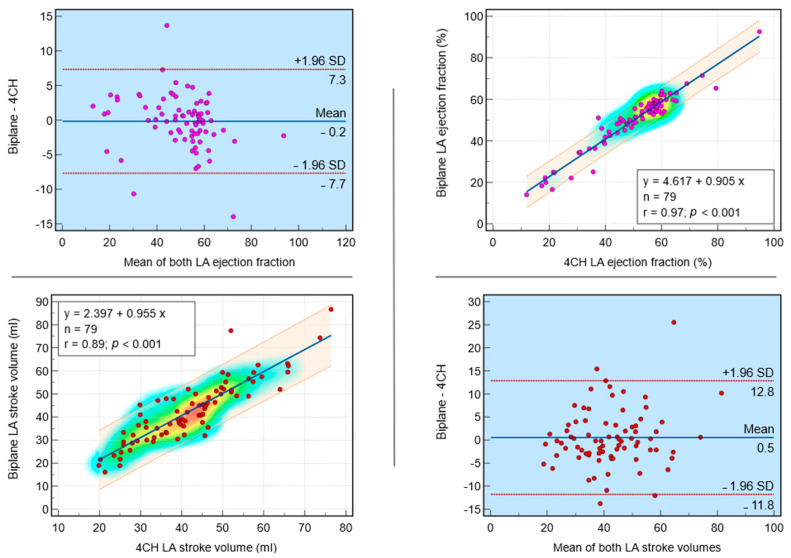
Post-bias correction agreement investigation in the validation cohort (*n* = 79): Bland–Altman plot agreement and correlation between biplane and single plane methods of left atrial (LA) ejection fraction (EF) and stroke volume (SV) assessment. Both Bland–Altman and regression analyses demonstrate the agreement of biplane versus single plane measurements for these two variables.

Comparison between the biplane and single plane post-bias correction methods for measuring LA volumes are demonstrated in Table 2. Bland–Altman plots illustrating the agreement between biplane and single plane methods are shown in Figure 4 and Figure 5. In a comparative analysis of biplane and single plane analysis methods, for biplane LA maximum volume, the arithmetic mean difference was −2 mL (95% CI: −20.4 to 16.5 mL, *p* = 0.05), indicating a slight underestimation by single plane, but this difference was not statistically significant. Similarly, for biplane LA minimum volume, the mean difference was 0.5 mL (95% CI: −11.1 to 12.1 mL, *p* = 0.47), suggesting a slight underestimation by single plane, but again, this difference was not statistically significant. For biplane and single plane LA SV and LA EF, the mean differences were 0.5 mL (95% CI: −11.8 to 12.8, *p* = 0.47) and −0.2% (95% CI: −7.7 to 7.3, *p* = 0.75), respectively, indicating no significant differences between the two methods. These findings suggest that the two methods are comparable for measuring LA maximum volume, LA minimum volume, LA SV and LA EF.

#### 3.3.4. Receiver Operator Characteristics

**Figure 6:**
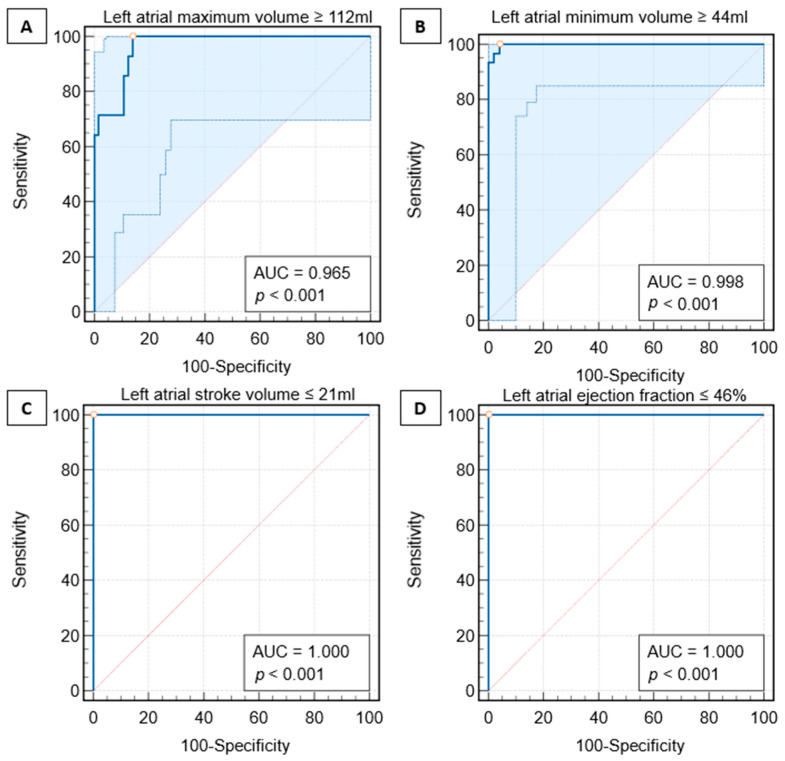
Receiver operating characteristic (ROC) Curves for left atrial (LA) parameters in predicting biplane cutoffs of abnormal values using the upper limit of normal values. [(**A**) maximum volume ≥ 112 mL, (**B**) minimum volume ≥ 44 mL, (**C**) stroke volume ≤ 21 mL, and (**D**) ejection fraction ≤ 46%]. Each panel displays the sensitivity (*y*-axis) against 100-specificity (*x*-axis). All *p*-values are less than 0.001, indicating significant predictive power for all metrics.

The area under the curve (AUC) for the single plane method to predict biplane method cutoff of LA maximum volume of ≥112 mL was (AUC = 0.97, *p* < 0.001), indicating a high level of accuracy in distinguishing between normal and abnormal states. Moreover, the single plane method to predict the biplane method cutoff of LA minimum volume of ≥44 had an even higher AUC = 0.99 (*p* < 0.001), suggesting excellent accuracy. Additionally, single-plane-derived LA parameters to predict biplane LA SV of ≤21 mL had an AUC of 1 (*p* < 0.001). Finally, single-plane-derived LA parameters to predict biplane LA EF of ≤46% had an AUC of 1 (*p* < 0.001), indicating ideal accuracy (Figure 6).

The results suggest that these variables are highly predictive and could be useful in disease diagnosis or prognosis. The standard errors were 0.02 mL for LA maximum volume, 0.002 mL for LA minimum volume, 0 mL for LA SV and 0% for LA EF. The 95% confidence intervals ranged from 0.89 to 0.99 mL for the LA maximum volume, 0.95 to 1.0 mL for the LA minimum volume, 0.95 to 1.0 mL for the LA SV and 0.95 to 1.0% for the LA EF. These findings provide robust evidence for the reliability of these variables in the study context.

## 4. Discussion

This study aimed to investigate if the single plane method for LA volume assessment on CMR is as good as the biplane method for clinical assessment. We observed that both methods had an excellent association with each other with some systematic biases. After correcting for biases, both methods are comparable and in strong agreement with each other. When we looked at clinically relevant cutoffs in the validation cohort, the single plane method demonstrated a high area under the curve for diagnosing a dilated left atrium, as per the biplane method. 

Previous studies used CMR to compare LA indices derived from biplane and single plane methods [17]. Tao et al. [17] demonstrated that the single plane tissue tracking CMR underestimated LA volume and strain compared to the biplane method. To compensate for that, they used regression equations to further quantify LA indices from a single plane. They found no significant differences between single plane and biplane methods in all LA volume and strain parameters. They concluded that the single-plane-derived CMR method is reproducible and as accurate as the standard biplane method. The relevant findings in this study provided the foundation we used to evaluate our study’s data further. However, despite demonstrating consistency and agreement between single plane and biplane methods, their method did not appear to account for any systematic bias. The single plane method underestimates volumes systematically; therefore, our study used a systematic correction factor to correct it to the biplane method. Given that their method does not routinely incorporate LA volumetric assessment, our approach offers broader applicability compared to tissue tracking or strain analysis. Moreover, our segmentation analysis method was completely automated, making it more feasible than tissue tracking CMR.

LA volume is considered more reproducible than left atrial strain [22,23] as it is less affected by the acoustic window and image quality, and it is based on geometric assumptions that are less complex than those required for strain analysis [24]. The reproducibility of left atrial strain can be affected by the load dependency of the parameter and the wide range of normal values, which can introduce variability in the assessment of left atrial function, particularly in patients with normal left ventricular EF [25]. It is also worth noting that several software vendors have different strain algorithms, which further prohibit its broader clinical adoption. Meanwhile, the area-length method for volumetric assessment is already largely used and is available in many cardiac-specific and generic applications. In summary, the more reproducible nature of LA volume measurements can be attributed to the established and standardised techniques for volume quantification, less sensitivity to image quality and operator dependency, and the geometric simplicity of the measurements compared to the complexity of strain analysis.

In routine clinical practice, the use of the biplane method for computing LA volume can be hindered by several challenges. These include temporal misalignment between the four-chamber and two-chamber cines, and improper planning of either cine. A well-executed four-chamber cine is of significant clinical value as it facilitates a comparative analysis of the LA and other chambers. Moreover, it enables the assessment of the pericardium, subcutaneous adipose tissue and descending aorta. Therefore, in situations where high-quality cines in other views are unavailable due to arrhythmias or other issues, a four-chamber analysis can prove to be invaluable. This study enhances the clinical applicability of four-chamber analysis by calibrating several methods for LA volume assessment. Consequently, this research significantly advances the field.

### Limitations

The four-chamber CMR analysis depends on acquisition; if wrongly planned, it can lead to under or overestimating LA volumes. However, we had clear acquisition protocols, likely reducing this error. We used already developed artificial intelligence (AI) deep-learnt contours to derive both biplane and single plane volumes. There is room for error in the accuracy of the AI contours; however, in our in-house testing, this has been minimal. Even though both derivation and validation datasets include a broad range of left ventricular impairment patients, we did not include congenital studies, which may limit our methods’ more general clinical applicability. Future studies are needed to test the prognostic relevance of our single plane method to measure LA volume.

## 5. Conclusions

LA volumetric and functional assessment by the single plane method has a systematic bias compared to the biplane method. After applying the bias correction factors, single plane four-chamber-derived LA volume and function are comparable to the biplane method.

## Figures and Tables

**Table 1:** Left atrial volumetric measurements by biplane and single plane analysis methods of the derivation cohort (*n* = 100).

Derivation Cohort	Biplane Method	Single Plane Method	Bias	SD of Bias	*p*
LA maximum volume, mL	96 ± 29	86 ± 30	−9	9 *	<0.01
LA minimum volume, mL	50 ± 27	42 ± 26	−8	6 *	<0.01
LA stroke volume, mL	45 ± 13	45 ± 13	−1	6	0.27
LA ejection fraction, %	50 ± 14	55 ± 15	−5	4 *	<0.01

Mean ± standard deviation. * The standard deviation of the bias has been used to correct the systematic bias. Abbreviations: LA, left atrium; SD, standard deviation.

**Table 2:** Left atrial volumetric measurements by biplane and single plane post-bias correction analysis methods of the validation cohort (*n* = 79).

Validation Cohort	Biplane Method	Single Plane	Bias	*p*
LA maximum volume, mL	90 ± 34	92 ± 34	−2	0.06
LA minimum volume, mL	48 ± 30	47 ± 30	0.5	0.47
LA stroke volume, mL	43 ± 14	42 ± 13	0.5	0.47
LA ejection fraction, %	50 ± 14	50 ± 15	−0.2	0.69

Mean ± standard deviation. Abbreviations: LA, left atrium; SD, standard deviation.

## Data Availability

The datasets generated and analyzed during the current study are not publicly available. Access to the raw images of patients is not permitted since specialized post-processing imaging-based solutions can identify the study patients in the future. Data are available from the corresponding author upon reasonable request.

## Abbreviations

AF	atrial fibrillation
AUC	area under the curve
CMR	cardiovascular magnetic resonance
EF	ejection fraction
LA	left atrium
LVFP	left ventricular filling pressure
ROC	receiver operator curve
SD	standard deviation
SV	stroke volume
